# Parameterisation and Prediction of Intra-canal Cochlear Structures

**DOI:** 10.1007/s10439-023-03417-5

**Published:** 2024-01-02

**Authors:** Joshua Thiselton, Tania Hanekom

**Affiliations:** Bioengineering, Department of Electrical, Electronic and Computer Engineering, University of Pretoria, Lynnwood Road, Pretoria, 0002 Gauteng South Africa

**Keywords:** Anatomic model, Cochlear duct, Cochlear implant, Anatomic landmarks

## Abstract

Accurate 3D models of the cochlea are useful tools for research in the relationship between the electrode array and nerve fibres. The internal geometry of the cochlear canal plays an important role in understanding and quantifying that relationship. Predicting the location and shapes of the geometry is done by measuring histologic sections and fitting equations that can be used to predict parameters that fully define the geometry. A parameter sensitivity analysis is employed to prove that the size and location of the spiral lamina are the characteristics that most influence current distribution along target nerve fibres. The proposed landmark prediction method more accurately predicts the location of the points defining the spiral lamina in the apical region of the cochlea than methods used in previous modelling attempts. Thus, this technique can be used to generate 2D geometries that can be expanded to 3D models when high-resolution imaging is not available.

## Introduction

The functioning of cochlear implants (CI) is dependent on the biophysical interface between the electrode array and the surrounding cochlear tissue. Computational models provide a mechanism to probe the complexities of this interface. These models rely on a three-dimensional (3D) description of the relationship between the electrode array, through which electrical stimulation is applied, and the surrounding tissue, which provides a medium for current conduction and contains the target neural elements.

The models can be used to predict the distribution of electrical stimulation current and the consequent neural excitation behaviour, thereby forming the link between electrical stimulus and neural response [1]. The models may also serve as a 3D visualisation tool that shows the relative locations of structures that may be difficult to view with standard imaging techniques. This can provide insight to improve surgical insertion of the electrode array by visualising the shape of the cochlear canal [2]. Post-operatively, it can also be used to view the relative positions of the electrode surfaces and spiral lamina which can assist with mapping procedures.

The two characteristics of a computational model that provide a realistic representation of a system are accuracy and solvability. Accuracy is the complexity of the anatomical representation of geometrical volumes as well as the division of the geometry into volumes of similar electrical properties. Solvability is related to the time taken by a numerical solver (in this case, COMSOL) to converge to a solution with a given set of boundary conditions. However, the complexity of natural tissues and interpersonal anatomical variation [3, 4] leads to a trade-off between the two. Constructing a volume conduction (VC) model representing the complex anatomy of the cochlea involves the discretisation of volumes of tissues with similar electrical properties. However, if all structures were to be included for the sake of accuracy, the large size differential among structures, important to the electrical behaviour of the system, may render an unsolvable mesh. An informed decision on the simplification of geometrical representations for the purpose of meshing, and therefore solvability, must thus be made.

While studies on generic models of human cochleae are fairly common [1], person-specific models of live subjects are less so. This is primarily because most modelling techniques rely on high-resolution imaging to extract the volumes or landmarks that define the anatomical structure. Generating user-specific models can be done via one of two techniques. Landmarking involves the identification of notable points on two-dimensional (2D) images that can be joined by a lattice of predefined lines to form areas [5]. The 2D images can then be extrapolated to 3D using modelling software. Segmentation involves the discretisation and filtering of the colour gradient on either 2D or 3D images. The different colour bands can then be assigned different volumetric properties [6]. Landmarking is less dependent on clear boundaries between tissue types and can be approximated by human observers, meaning that it is more suited for images with poor clarity [7].

Most high-fidelity geometry quantification techniques utilise micro-computed tomography (µCT) images [8, 9], dissection and photography or more advanced techniques like scanning thin-sheet laser imaging microscopy [10] as they provide high-resolution images with the required detail to extract accurate volumes. However, they can only be used on cadavers due to high radiation output or cranial bone dissection. This leaves computed tomography (CT), X-ray and magnetic resonance imaging (MRI) as the only viable options when attempting to model cochleae of live subjects. MRIs produce large artefacts for subjects with CIs and X-rays can only provide a single cross-sectional image. Thus, CTs are the best available option, even though they are only able to show the outlines of the cochlear canal. The construction of a high-fidelity 3D cochlear model requires detail of internal structures not visible on CTs.

While there are several methods that attempt to accurately predict the shape of the cochlear canal [11, 12] and a few that place the spiral lamina [8, 13, 14], there are no statistical or geometrical models that attempt to define the anatomical structures within the cochlea via anatomical references. One previous attempt used a transformation and fitting (morphing) of a template canal cross-section to a known canal shape and size [7]. Another used a statistical shape model to define a relationship between known shapes on $\mu$CTs to predict locations on CTs, but only attempted to find the spiral lamina [15]. Basic landmarking has been done to define the curvature of the canal and the structures within [16], however, the internal geometry was estimated without anatomical reasoning.

A number of anatomical structures are important for predicting current distribution throughout the cochlear volume. It is known that both the basilar membrane and Reissner’s membrane provide high-impedance barriers to current existing [17] along the cochlear duct. Also, the impedance of the duct, which will determine the magnitude of the current inside the duct, will be influenced by the location of both membranes [18]. The spiral lamina location is important for the same reason, but also because this structure contains the peripheral axons of the nerve fibres to be stimulated. Neural degeneration is another concern for CI users [19], meaning that excitation at the organ of Corti is seldom possible and often occurs at the spiral ganglion level in the modiolus [20, 21]. The modiolar wall of the scala tympani (and the scala vestibuli if a scala vestibuli insertion or a translocation of the electrode from the scala tympani to the scala vestibuli occurred), is an important boundary that delineates the region where spiral ganglia exist. The location of this boundary therefore has an important effect on nerve excitation [22]. The location of the spiral ligament is also important as current is known to leak among the scala tympani, scala media and scala vestibuli through this structure [23].

This study aims to provide a parametric model of the internal structure of the cochlea through reference to measurable landmarks. The model is derived from landmarks that are measured on mid-modiolar histologic sections (HS) of the cochlea. Because these landmarks form the basis for the prediction of the geometry of the internal structures, the resulting model should be a more accurate representation of a CI recipient’s cochlea. The model is compared with the morphing method described in [7] and is validated against an independent set of HSs. A parameter sensitivity analysis is performed to investigate which structures have the greatest effect on potential distributions resulting from intra-cochlear stimulation.

## Method

Parameterisation of the internal geometry of the cochlea is an expansion of the work in [24]. They developed a method to identify, measure, or predict the cochlear canal boundary landmarks (*LS, SLS, SS, SMS, MSVS, STS, IMS, IS*, and *ILS*) and their associated spirals for individual CI recipients from low-resolution clinical images. However, the model lacks the internal structure of the cochlea which is not visible on low-resolution images. The construction of the parametric model involves the definition of an inner-structure template geometry referenced to the existing boundary landmarks, a description of relevant parameters, selection of appropriate source data and quantifying the parameters using the source data. Finally, equations describing the parameters are derived using curve fitting of the quantified source data.

### Geometry Parameterisation

**Fig. 1:**
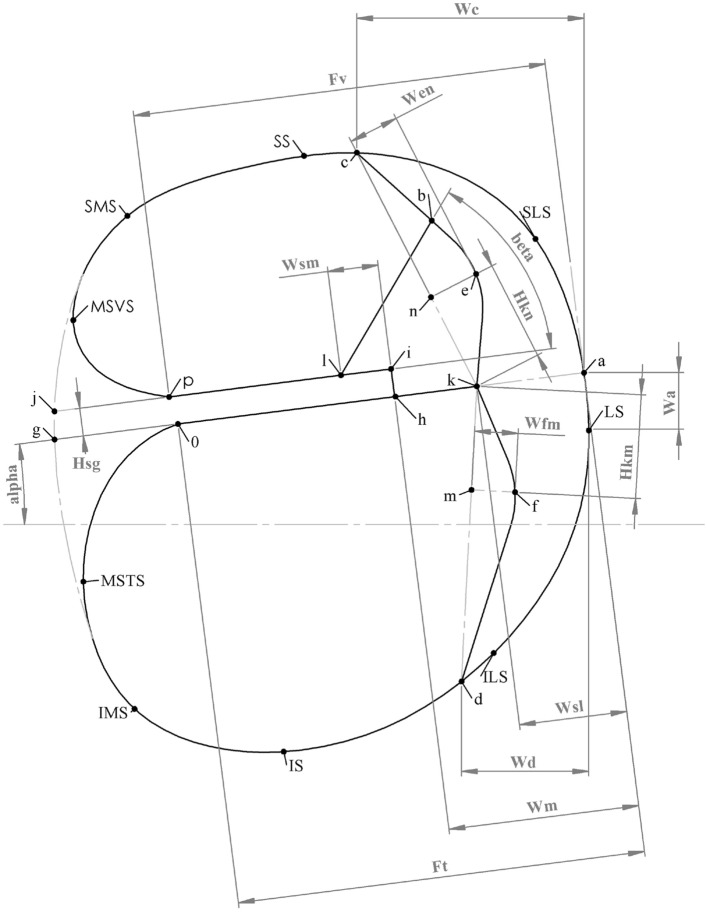
Dimensions used to define the cross-section of the cochlear duct. Landmarks *LS, SLS, SS, SMS, MSVS, MSTS, IMS, IS* and *ILS* define the boundary of the cochlear canal. The landmarks *a* to *n* are placed in the measuring process to derive the dimension values

To place the landmarks that define the internal geometry, described in Table 1, high-resolution images that show the internal structure of the cochlea are required. HSs were chosen as it is possible to identify and place the boundary and internal structure landmarks precisely and accurately. Defining the reference geometry as seen in Fig. 1 involved segmenting an HS according to different tissues and known anatomical boundaries. The regions within the traced shapes are treated as homogeneous areas. The thin regions [basilar membrane, Reissner’s membrane, stria vascularis, and bone in the spiral lamina (SL)] are simplified to lines. Their volumetric nature can be simulated as contact impedances in VC applications. Small structures, such as the organ of Corti and tectorial membrane, are combined with surrounding structures as a means to improve solvability as part of the trade-off with geometrical accuracy.

**Table 1:** Description of the interior geometry landmarks shown in Fig. 1

	Description
*a*	Projection of BM meeting the curve representing the canal wall
*b*	Intersection between Reissner’s membrane and spiral ligament
*c*	Intersection of the spiral ligament in the scala vestibuli and the canal wall
*d*	Intersection of the spiral ligament in the scala tympani and the canal wall
*e*	Most lateral point of the spiral ligament in the scala vestibuli
*f*	Most lateral point of the spiral ligament in the scala tympani
*h*	Intersection of the SL and BM
*i*	Apical, lateral point of the SL
*k*	Intersection of BM and the spiral ligament
*l*	Intersection of Reissner’s membrane (RM) and the SL
*p*	Apical intersection of the canal wall with the SL
*o*	Basal intersection of the canal wall with the SL

To derive equations for the internal structure (which is not visible on clinical images), the mathematical relationship among the various structures needs to be determined through lines and angles referenced to a fixed point. The parameters selected to fully define the internal geometry are described in Table 2. The lateral spiral (LS in Fig. 1) is chosen as the reference point based on the assumption that it can be accurately identified on clinical images. Defining parameters in relationship to other boundary points is more likely to result in error stacking as they are not always clearly distinguishable. A combination of straight lines and fourth-order Bézier curves were used to approximate the boundaries between different tissue areas. For example, a straight line is used to represent the BM while a Bézier curve is used to represent the wall of the cochlear canal. Straight lines can either be defined by a point and an angle or two points. Due to using a single point (LS) as a reference for all parameters, the point and angle method is preferred. However, the second point is still needed for the landmarking geometry definition technique. It is found through the intersection with other structures. For example, the line *a–g* is defined by an angle (alpha) and a point (*a*). Point *g* is then found where the straight line intersects with the outline (*LS* to *ILS*).

**Table 2:** Description of the parameters used to generate the equations given in Table 5

	Description
alpha	Angle of the BM relative to horizontal
Wa	Vertical distance between (*LS*) and *a*
Wm	Distance between the organ of Corti and the canal wall, parallel to the SL
Hsg	Thickness of the SL
Ft	Distance between *a* and o along the SL
Fv	Distance between *a* and p along the SL
Wsl	Width of the scala ligament at the BM, parallel to the SL
Wc	Horizontal distance between *a* and *c*
Wd	Horizontal distance between *a* and *d*
Hkm	Distance between *k* and f parallel to the line projection *k–d*
Wfm	Perpendicular distance between the line projection *k–d* and *f*
Hkn	Distance between *k* and e parallel to the line projection *k–c*
Wen	Perpendicular distance between the line projection *k–c* and *e*
Wsm	Distance along the spiral lamina between the organ of Corti and RM
beta	Angle of Reissner’s membrane relative to the SL

“Vertical” is the direction parallel and “horizontal” is the direction perpendicular to the modiolar axis

Person-specific anatomical variations in the geometry of the internal structures of a model will occur as a result of the variation in the canal boundary defined by LS and the other eight boundary landmarks.

### Source Data Selection

Mid-modiolar histologic cross-sections of the cochlea were obtained from the internet, of which 27 were selected [25–36], based on clarity, correct orientation, and absence of damage to internal structures. The appearance of the first two turns of the canals was used as a criterion for selection, as cochlear implant electrode arrays are seldom inserted beyond 540° from the round window. Landmarks were measured on the HSs to provide the data that will be used to generate the equations. The criterion for correct orientation is that the cochlea must be sectioned along the mid-modiolar axis in such a way that the internal detail of the first two turns of the cochlear canal is clearly identifiable.

The two disadvantages of HSs are that a slight distortion occurs in the preparation process and that data have to be measured from a single cross-section. For cochlea sectioning during the preparation process, the error in landmark deviation caused by the distortion is negligible [34]. However, a single section through the cochlea only provides a single reference for its 3D structure and may not contain information about the rotation angle at which the section was taken.

Corrections are made to mitigate the error for mid-modiolar sections taken at unspecified rotation angles. The parameters derived from the landmark measurements (Table 2) are normalised to the width of the most basal cochlear canal so that the parametric equations are independent of rotation angle. They are instead dependent on a dimension of the cochlear canal identifiable on low-resolution images. Any further error introduced by a possible rotation offset is assumed to be negligible. Another source of error is misalignment with the modiolar axis, which may cause distortion, e.g. elongation of the structures. This is minimised through the HS selection process, though slight elongation in the basal region and distortion in the apical region may still be present. If the mid-modiolar axis of the HS is not aligned with the coordinate system, the image is rotated using the angle of the spiral lamina measured relative to the horizontal axis of the image as a guideline. This involves iteratively rotating the HS images until alpha (from Fig. 1) has the lowest residual when fitting a straight line through the measured angles as a function of cochlear angle.

### Measurements

**Fig. 2:**
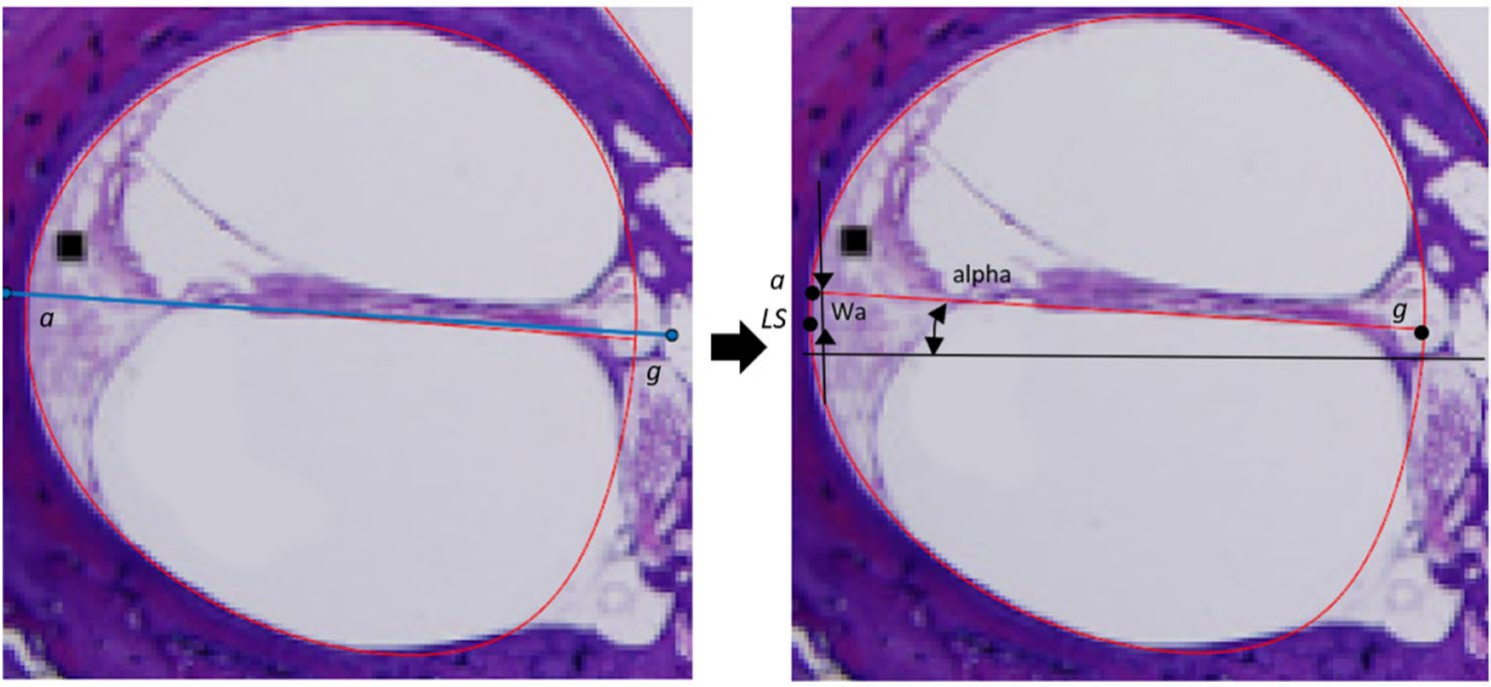
Landmark placement is done by placing tracing lines (blue) on HSs (left). These are used to create boundaries (red) between different tissue areas (right). The basilar membrane (BM) is placed across the cochlear canal and aligned with the visible membrane and spiral lamina sections. The placement is used to determine the Wm and alpha measurements. The HS is taken from [28]

**Fig. 3:**
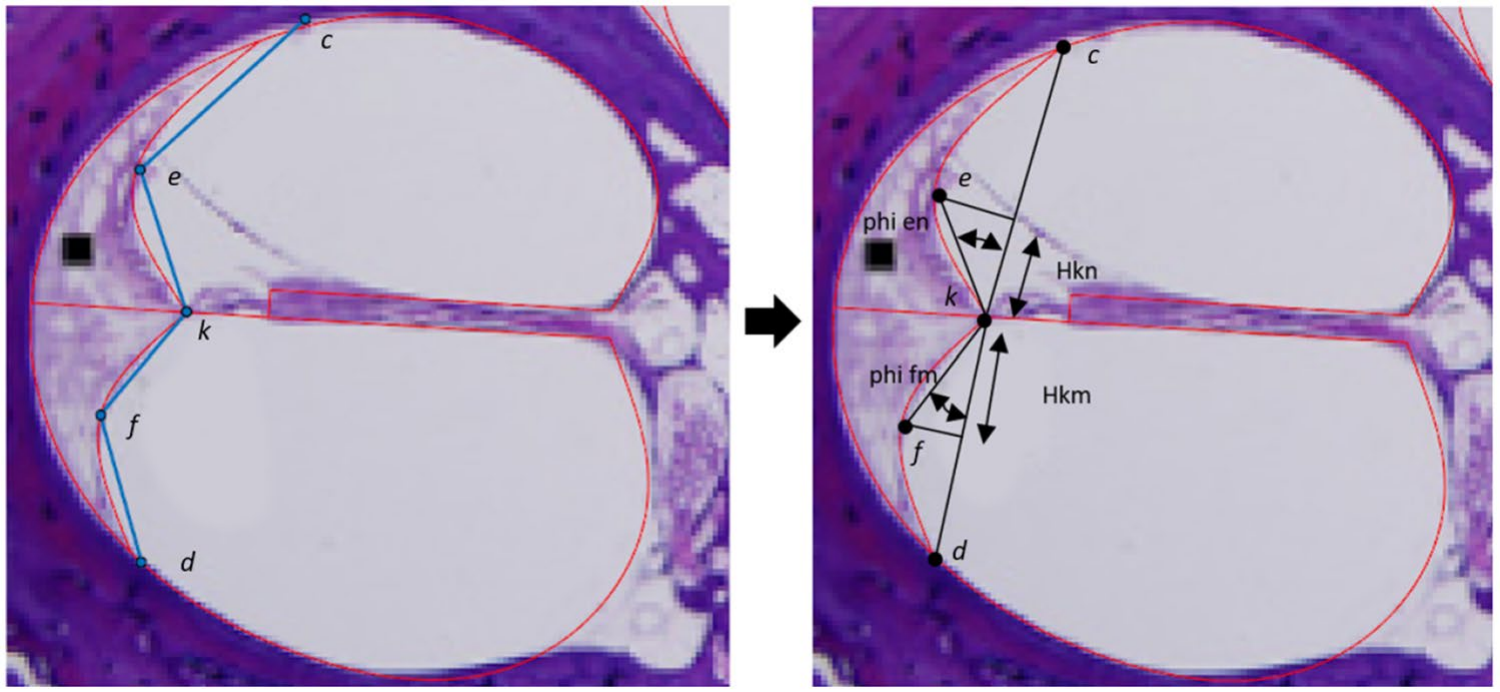
The spiral ligament boundaries are placed using five landmarks that approximate the ends and the middles of the curves that make up the boundaries with the cochlear canal (left). A fourth-order Bézier curve (red) is plotted with the landmarks, which are also used to measure Hkm, Hkn, $\phi _{en}$ and $\phi _{fm}$ (right). The HS is taken from [28]

The 27 cross-sections were measured not by finding landmarks and drawing lines among them, but by placing pseudo-landmarks on the visible curve, fitting straight lines to them, and selecting the landmarks as the points at which the lines intersect each other or the boundaries as explained in the previous section. Pseudo-landmarks are points placed between anatomical or mathematical landmarks that are used to define curves. Figure 2 illustrates this process for the basilar membrane. The placement of landmarks is shown on the left and the quantification of the parameters is presented on the right. Figure 3 shows how the curves for the spiral ligament (lines *c–e–k* and *k–f–d*) are fitted using two fourth-order Bézier curves defined by the six aforementioned points. The measurements are then extracted from the landmark relationships and normalised according to the basal canal, specifically the distance between LS and the average point between the MSVS and MSTS landmarks. The canal width is the normalising distance as this is a dimension that can be measured on most CTs across all mid-modiolar sections. Values such as cochlear length or height cannot be used as they cannot be extracted from the HSs due to having only a single section of unknown angular relation to the length or width axis.

### Equation Derivation

**Fig. 4:**
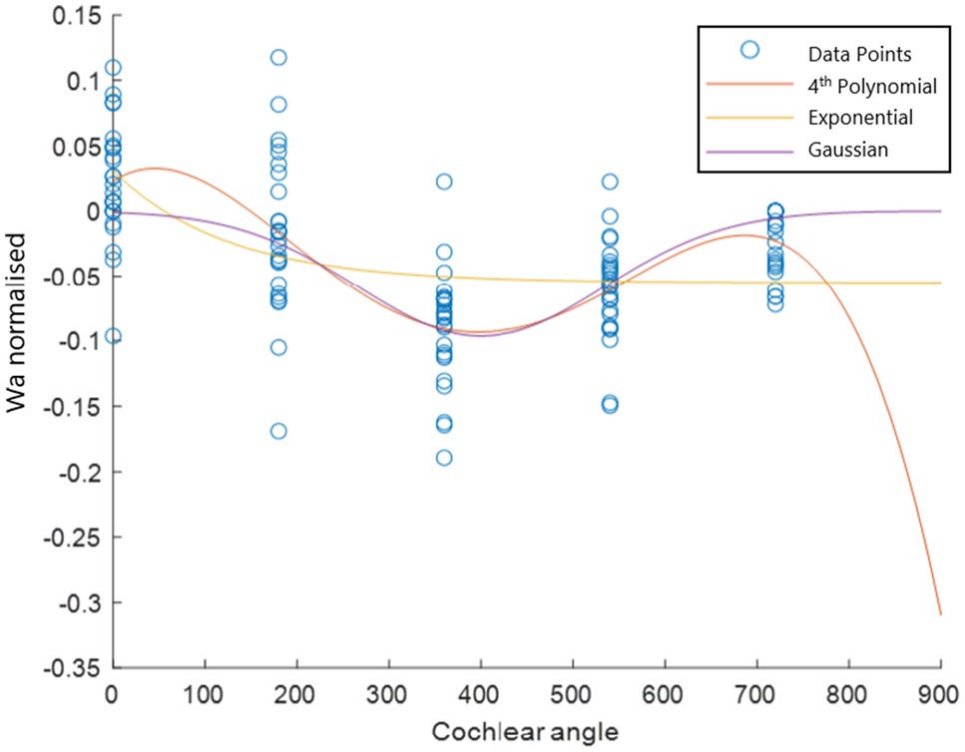
The measured values of Wa are shown with the fourth-order polynomial, exponential and Gaussian curves fitted to that data. Some 3D cochlear models include the apical region of the cochlear (up to 1080°), meaning that extrapolation may be needed. Some fits, such as the fourth-order polynomial that typically produces unrealistic extrapolation, should be avoided, whereas others, such as the Gaussian curve, may produce more realistic extrapolations, even if accuracy cannot be proven

The equations were derived by taking the measurements of the 27 cross-sections and fitting polynomial curves, up to fourth order, and exponential curves, up to second order, to the measured data. Outliers were removed using a 20–80 percentile window, and then a root mean square error (RMSE) value was determined for each curve. The equation with the lowest RMSE was selected. An example of the measured data for Wa and three of the fitted curves are shown in Fig. 4. The Gaussian equation (purple curve) was selected as it has the lowest residual of 0.0019 compared to the fourth-order polynomial’s 0.0021 or the exponential’s of 0.0025.

The equations were then used to develop cross-sections using the LS points for five validation data sets [26–28, 36]. In total, 32 HSs were used in this study comprising 27 modelling sections and five validation sections. The RMSE for all the landmarks in the validation sections was calculated using Eq. 1. The subscript *m* indicated the measured value and *p*, the predicted one.

1 $$\begin{aligned} \text {RMSE} = \sqrt{\sum \frac{\vert (x_m-x_p)(y_m-y_p)\vert }{n}} \end{aligned}$$

### Validation of Landmark Prediction Method

[7] used a template morphing method to add internal structures to person-specific models of the cochlea. This method involves taking a generalised internal structure geometry and fitting it to the boundaries of the cochlear canal as determined from clinical images of a CI recipient. A comparison of the proposed method with the template morphing method, using the same HSs, was used as validation. The generalised geometry for the template morphing method was extracted from the most basal canal of one of the measurement HSs [26]. This generalised internal structure geometry was then fitted (morphed) to the box defined by the measured *IS*, *LS* and *SS* landmarks, as well as the midpoint between *MSVS* and *MSTS* by scaling, translating, and mirroring it. The template was morphed onto each of the sections through the first two turns of the cochlea on each HS in the validation set. The morphing method landmarks *a* to *o* were then compared to the parameter generation landmark locations via the RMSE method.

### Parameter Sensitivity

**Fig. 5:**
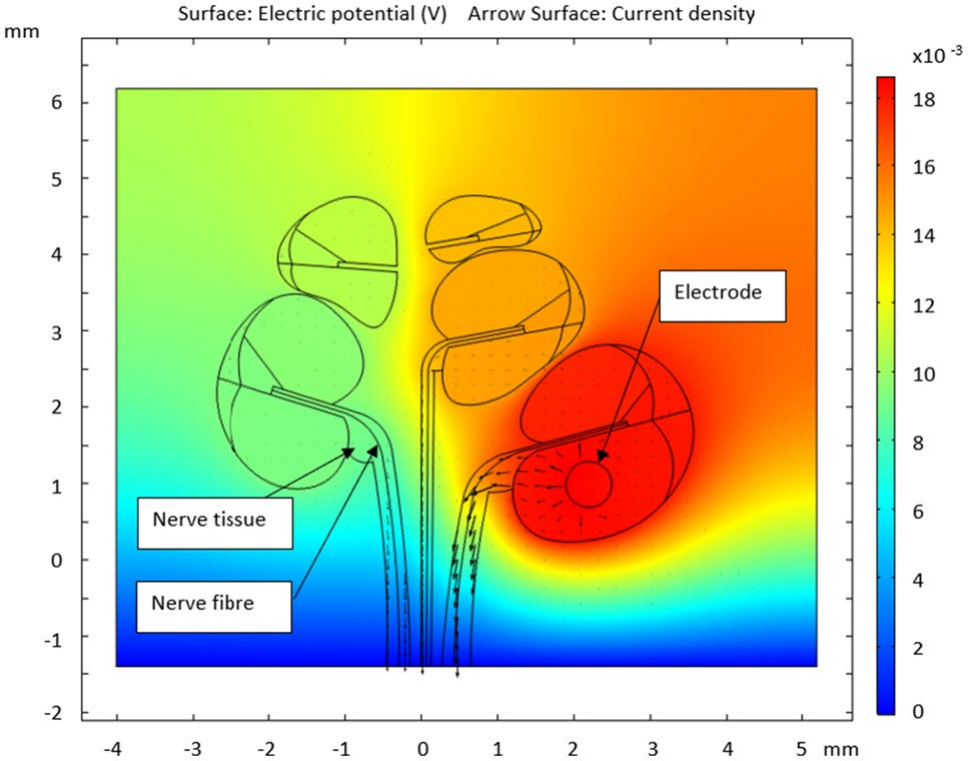
Geometry with an overlay of the potential distribution and current density for a single iteration of the sensitivity analysis. The unit of the colour bar is Volt. The current density lines show the tendency for the current to leave the cochlea via the nervous tissue

It is necessary to understand the impact that geometrical errors will have on model outputs. This is achieved through a finite element method (FEM) parameter sensitivity test that assesses the impact that varying each parameter has on the potential distribution at the nerve fibre. The procedure involves constraining all the parameters except the one that the particular study focuses on. The range, for which the parameter is varied, is limited by the geometry of the surrounding structures and the 20th–80th percentile of the measured data. For example, alpha is only less than 0^∘^ in the lower 20% of the measured data and exceeds 15^∘^ in the 80% and Wm can never be less than Wsl and only exceeds the 80th percentile at 0.6 mm. The 2D geometry used for the analysis is shown in Fig. 5, in which a single cross-section is selected and extended to contain both a nerve tissue area and an electrode in the scala tympani of the basal turn. A single nerve fibre is plotted within a region assigned as nerve tissue for the three most basal half-turns.

The electric FEM is set up as a purely resistive model (the permittivity of all the materials is 1) to solve Eqs. (2) to (4). $\textbf{J}$ is the current density, $Q_{j,v}$ the location-specific charge, $\textbf{J}_e$ an externally generated current density, $\textbf{E}$ the electric field intensity, $\sigma$ the electrical conductivity and *V*, the electric potential.

2 $$\begin{aligned}{} & {} \nabla \cdot \textbf{J} = Q_{j,v} \end{aligned}$$

3 $$\begin{aligned}{} & {} \textbf{J} = \sigma \textbf{E} + \textbf{J}_e \end{aligned}$$

4 $$\begin{aligned}{} & {} \textbf{E} = -\nabla V \end{aligned}$$

A stationary solving function is applied, meaning that the predictions shown are the static values achieved once the potentials have converged after an initial condition of 0 V throughout. The input function is a constant 2 mA applied to the modiolar facing boundary of the cross-section of a half-band electrode as found in the Cochlear Contour Advance array.

The external model domain was a square, approximately four times the width of the cochlea with the cochlea in the centre. The lower boundary of the model domain was used as reference. The nerve tissue was extended along the modiolar axis down to the boundary of the model domain, which is a simplification of the actual nerve trajectory. The conductances for the homogeneous areas in the simulation are given in Table 3. The model also assigns contact impedances to some boundaries, the values and thicknesses of which are given in Table 4. The temporal bone contact impedance was placed around the outside of the cochlear canal and between the scalae and the nerve tissue within the spiral lamina. The simulation is solved using COMSOL’s AC/DC module to generate the potential distribution throughout the model domain.

**Table 3:** Conductivity values used in the sensitivity analysis

Material	Conductance (S/m)
Modiolar bone	0.016
Nerve	0.33
Electrode silicon	$1\times 10^{-7}$
Scala vestibuli/tympani	1.43
Scala media	1.67
Spiral ligament	1.67

**Table 4:** Contact impedance values used in the sensitivity analysis

Material	Conductance (S/m)	Location	Thickness (mm)
Temporal bone	0.0334	Around each canal, including lining the spiral lamina	0.02
Basilar membrane	0.0126	Between the scala media and tympani	0.005
Reissner’s membrane	0.0001	Between the scala media and vestibuli	0.002
Stria vascularis	0.005	Along the lateral wall of the scala media	0.01

## Results

### Parameter Equations

The equations developed for the parameters in Fig. 1 are shown in Table 5. The majority of dimensions (10 out of 16) were best represented by an exponential function. Four dimensions (Hsg, Ft, Wc and beta) were best represented by third-order polynomial equations. These are shown with a set of centring values to translate and scale the polynomial curves to fit the data. The Wsm and BM parameters were best approximated with straight-line fits. This corresponds to basilar membrane measurements performed on human cochleae [37].

**Table 5:** Equations pertaining to the dimensions shown in Fig. 1 as a function of cochlear angle ($\theta$) in degrees

		Equation	Centring $[c_1,c_2]$
Alpha	=	$0.11423\times \exp {(-34\times 10^{-6}(\theta -250)^2)} + 0.0977$	
Wa	=	$-0.09641\times \exp {(-2.8\times 10^{-5}(\theta -400)^2)}$	
Wm	=	$0.4162\times \exp (-0.0001(\theta + 200)^2) + 0.3524$	
Hsg	=	$-0.0013\theta _n^3 + 0.0017\theta _n^2 - 0.0016\theta _n + 0.0425$	[338.28; 242.26]
Ft	=	$-0.01562\theta _n^3 + 0.0433\theta _n^2 - 0.0217\theta _n + 0.759$	[339.11; 244.91]
Fv	=	$0.1391\times \exp (-8.5\times 10^{-3}\theta ) + 0.7666$	
Wsl	=	$0.2156\times \exp (-2.5\times 10^{-3}\theta ) + 0.01466$	
Bm	=	$2.0005\times 10^{-4}\theta +0.157$	
Wc	=	$-0.0243\theta _n^3 + 0.0482\theta _n^2 - 0.0484\theta _n + 0.2087$	[341.69; 241.6]
Wd	=	$0.0727\times \exp (-4.8\times 10^{-5}(\theta -400)^2) + 0.1086$	
Hkm	=	$0.22\times \exp (-1.7\times 10^{-5}(\theta +250)^2) + 0.4349$	
$\phi _{fm}$	=	$0.1996\times \exp (-2\times 10^{-3}\theta ) + 0.1344$	
Hkn	=	$-0.0631\times \exp (-0.01\theta ) + 0.4128$	
$\phi _{en}$	=	$0.2011\times \exp (-0.01\theta ) + 0.3964$	
Wsm	=	$-1.886\times 10^{-5}\theta + 0.074$	
Beta	=	$-0.0321\theta _n^3 + 0.0582\theta _n^2 + 0.1567\theta _n + 0.6729$	[341.22; 243.57]

Centring applies for polynomials where $\theta _n = (\theta - c_1)/c_2$

**Fig. 6:**
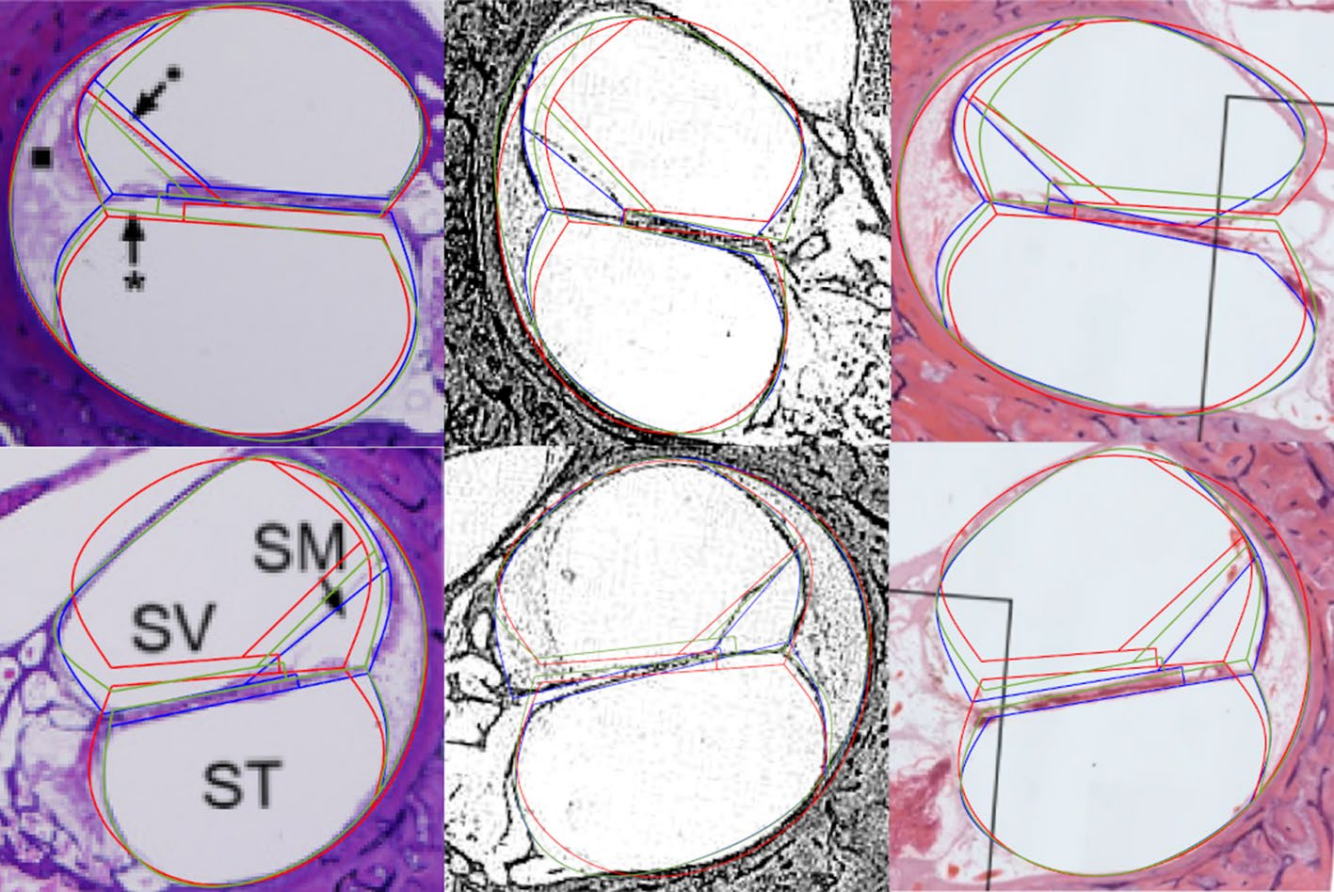
Measured (blue), predicted (green) and morphed (red) and geometries for the first half-turn ($0^\circ$: top row and $180^\circ$: bottom row) of HSs from [26, 28, 36]

**Fig. 7:**
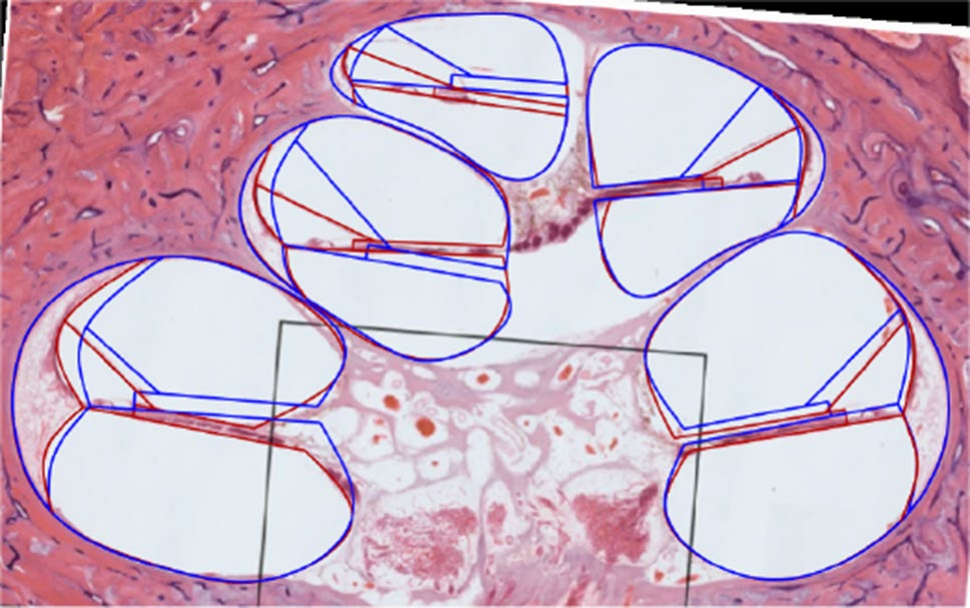
Measured (red) and generated (blue) geometries for the HS from [36]

The canals of the first half-turn ($0^\circ$ and $180^\circ$) of two of the five validation cross-sections are shown in Fig. 6. The geometries created with landmarks plotted with measurements (blue), the prediction equations from Table 5 (green) and the template morphing (red) are overlaid on the HS to allow visual comparison. A full cross-section is shown in Fig. 7 for the predicted geometry up to $720^\circ$.

The two sections shown in Figs. 6 and 7 are representative of the five validation sections. The figures suggest that the predicted location of the spiral ligament is similar to the actual location. The predicted length and height of the spiral lamina also correspond with the actual structures though the location deviates. The location of Reissner’s membrane deviates considerably from its true location. The predicted structures correspond better in the basal region, which may be attributed to the shape of the apical turns being less consistent among cochleae or due to apical regions in HSs being more distorted due to dissection and preparation error.

Table 6 shows a comparison of the RMSE values for the landmark prediction method and the control template morphing. The RMSE values are compared across each turn using a two-tailed *t*-test to generate the *p*-values given in the final row. A two-way ANOVA analysis was performed on each coordinate set for each landmark and the resulting *p*-values are given in the final column. The proposed method has RMSE values that are consistently lower than the control, however, the values for the first half-turn ($0^\circ$–$180^\circ$) are similar. This is because the shape of the basal turn of the cochlea is typically less affected by neighbouring turns than that of the more apical turns, resulting in less deviation of both the internal structures and the outline from a generic template. However, while the landmark prediction method becomes more inaccurate towards the apex, it is considerably better than the morphing method. The RMSE values suggest that the proposed method is more accurate throughout a $540^\circ$ turn model than the template morphing model. The lower basal turn ($0^\circ$–$180^\circ$) has a *p*-value of 0.36, meaning that the difference between the prediction methods is not statistically significant. However, the following three half-turns ($180^\circ$–$720^\circ$) all have *p*-values within a 98% confidence interval, indicating that there is a significant difference past the first half-turn.

**Table 6:** RMSE values for landmarks *a* to *p* for both the template morphing and the landmark prediction methods

	Template morphing				Landmark prediction				*p* value
	0°	180°	360°	540°	0°	180°	360°	540°	ANOVA
*a*	2.85	4.35	3.95	6.78	0.97	3.65	2.09	2.25	0.0052
*b*	4.55	11.61	17.23	8.43	5.89	8.43	18.90	9.34	0.9895
*c*	15.17	7.93	13.45	22.94	1.57	2.55	8.91	4.30	0.5915
*d*	8.60	5.19	16.73	9.89	9.87	4.83	9.84	7.17	0.0028
*e*	6.91	5.57	10.65	10.98	10.16	6.91	6.07	3.80	0.5518
*f*	7.11	4.37	7.80	8.23	2.78	3.76	7.67	3.72	0.7529
*h*	11.81	14.97	18.75	16.47	9.99	5.66	8.51	8.81	0.0367
*i*	11.19	14.55	18.00	15.89	7.52	5.41	8.70	9.70	0.1108
*k*	4.71	11.30	11.89	23.65	5.76	6.55	6.81	4.59	0.3809
*l*	10.49	11.15	17.53	18.21	9.85	6.98	9.47	11.68	0.1147
*o*	6.20	16.39	8.29	11.06	10.16	6.91	6.07	3.80	0.0657
*p*	6.65	17.25	12.69	9.99	6.55	21.18	9.18	14.73	0.1159
*p*-value (*t*-test)					0.3656	0.0188	0.0012	0.0067	

The *p*-value results for a two-way ANOVA analysis for each landmark are given in the final column. The *p*-value results for a two-tailed *t*-test analysis for all the landmarks at each turn are given in the final row

Of the landmarks, *a* and *d* have *p*-values lower than 0.01, meaning that there is a 99% confidence interval and that the difference in coordinate prediction is statistically significant. *h* and *o* lie around the 95% confidence interval and *l*, *i* and *p* lie around the 90% interval. The rest of the landmark prediction points are unlikely to significantly differ from the template morphing method.

### Parameter Sensitivity

**Fig. 8:**
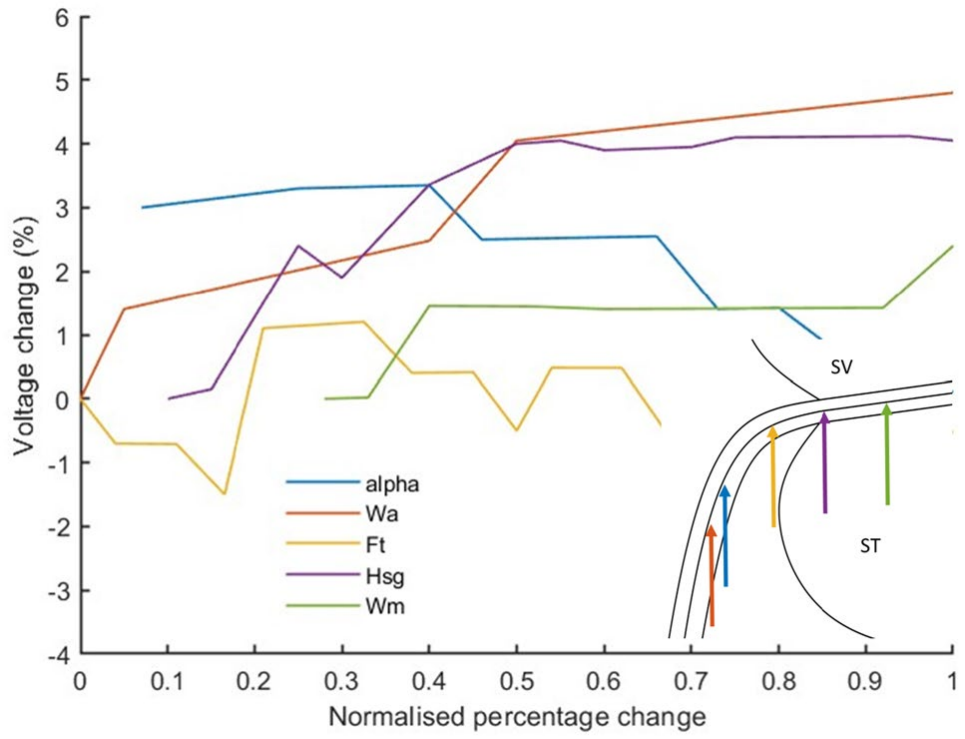
Locations along the nerve fibre that undergo the largest potential differences as a result of changes to the indicated parameters. A portion of the cross-section of the cochlear canal is shown, with the scala vestibuli (SV) and scala tympani (ST) indicated

The potential distribution along each single nerve fibre is extracted as a function of the nerve fibre length from the scala media to the point where the fibre passes the most inferior point of the basal turn. The point along the nerve fibre at which the maximum change occurs over the parameter sweep range is selected (shown in Fig. 8), and the potential at that point is given as a function of normalised parameter change.

From the results of the sensitivity analysis and based on the range of potential change, the parameters can be ranked in order of importance as Wa, Hsg then alpha followed by Wm and Ft. The other parameters have comparatively negligible impact, inducing less than 0.2% change. For this analysis, containing an electrode in the most basal turn, predictions for the two most apical fibres (refer to Fig. 5) were not considered as the potential changes were a factor of ten lower than those of the basal fibres.

## Discussion

The results of the FEM sensitivity analysis suggest that the parameter with the most notable impact on the potential distribution at the target nerve fibres is Wa, which is the vertical distance between the most lateral point (*LS*) and the projection of the basilar membrane (*a*). Alpha, ranked third in terms of importance, is the angle of the spiral lamina. The combination of these two describes the location of the spiral lamina. Hsg, the second most sensitive parameter, is the thickness (distance between *i* and *h*) of the spiral lamina. Wm is the distance between the most lateral point (*LS*) on the cochlear wall and the start of the spiral lamina (*h*). Thus, these two define the size of the spiral lamina. This suggests that the current loss through the organ of Corti and along the spiral lamina plays an integral role in nerve excitation. The finding also suggests that the spiral lamina is the most important internal structure that affects potential distributions at the nerve fibres in volume conduction computational models of the cochlea. Great care should thus be taken to correctly estimate the dimensions and location of this structure, especially when constructing user-specific models of the cochlea.

Ft defines the curve of the scala tympani as it joins the spiral lamina (landmark *o*). As the nerve pathway approximately follows the curve of the scala tympani walls, it is expected that this will impact neural excitation. These five parameters are a function of landmarks *a*, *h*, *i*, and *o*. *a*, *h* and *o* are more accurately predicted by the landmark prediction method than the template morphing method. This implies that the important characteristics of the spiral lamina and scala tympani boundaries are better encapsulated by the proposed landmark prediction method.

The location of *i* is important as it describes the thickness of the spiral lamina (Hsg). However, the difference between the results of the landmark prediction and the template morphing method is not significant (*p*-value of 0.11). A possible explanation for the smaller confidence interval could be that the thickness of the spiral lamina is difficult to accurately measure because of the limits on resolution relative to the small size of the structure, even on high-resolution images such as $\mu$CTs or HSs. It is also conceivable that the thickness of the spiral lamina does not vary significantly relative to the size of the other structures as suggested by the small coefficients in the equation of Hsg (Table 5). For this reason, either a constant value or the proposed equation for Hsg are considered reasonable approximations for the thickness of the spiral lamina.

As the rest of the landmarks describing the spiral ligament, Reissner’s membrane and the medial curve of the scala vestibuli are neither significantly different, nor have much impact on neural excitation, any method that approximates their location would be suitable. It would also be a reasonable assumption to exclude these structures if a simpler model is required.

It is known that people with cochlear implants have degenerated peripheral axons in the spiral lamina [19]. Figure 8 shows that the point along the nerve fibre, at which the maximum changes in the predicted potential distribution occur, coincides with the assumed location of the terminals of the degenerate fibres. This implies that models that assume neural degeneration may be particularly sensitive to the placement of landmarks that describe the shape and location of the spiral lamina.

To create high-fidelity volume conduction models of individual cochleae, it is important to include the inner structures that affect the distribution of the stimulus currents. Segmentation methods [11, 13] are not adequate to derive the inner structures of the cochlea from low-resolution image data of live CI users. Similarly, template morphing methods that include inner-structure details [7] do not accommodate person-specificity. The advantage of the proposed landmark-based inner-structure model over existing models is that it allows person-specificity to be included in both the shape of the cochlear canal and the geometry of the inner structures.

The limitations of this study are, firstly, that the orientation of the mid-modiolar HSs was unknown. While normalisation relative to the width of the most basal cochlear canal was used to mitigate this drawback, the accuracy of the landmark prediction method may be improved by deriving the equations from mid-modiolar HSs with known orientation. Similarly, increasing the sample size may improve the predictive power of the method especially if the cochlear class is known, allowing the equations to be derived based on cochlear taxonomy [38].

## Conclusion

Predicting the excitation behaviour of electrically stimulated auditory neurons is possible using computational models that mimic the physical and electrical characteristics of the cochlea. Although several models exist to describe the outer boundaries of the cochlear canal, the internal geometry of the live cochlea cannot be observed because of the limited resolution available in clinical imaging. An accurate description of the internal geometry of the cochlea is important as it affects the predicted spread of stimulation current. The proposed landmark prediction model provides a means of predicting the location of the internal cochlear structures. The model was derived from high-resolution 2D data obtained from HSs to describe the relationships among landmarks through a set of parametric equations referenced to the lateral-most point on the cochlear canal. This point is generally visible on low-resolution clinical images. The resulting 2D parametric description of the inner structures may be applied to successive mid-modiolar slices through a 3D representation of the cochlea to obtain a volumetric description of these structures. This means that the equations can be applied to improve the person-specificity of 3D models of the cochlea despite poor-resolution image data.

The landmark prediction method was shown to render more accurate predictions of the cochlear structures at more apical turns than the template morphing method. This is an important finding as the landmarks towards the apex are often more obscured than those at the base because of the smaller size and greater variation in the shape of the canal towards the apex. The method also provides an improvement over the template morphing method in predicting the location of the spiral lamina landmarks. It was shown that the shape and size of the spiral lamina have the greatest effect on current spread reaching the target neural fibres, suggesting that this inner structure is the most important to represent in person-specific volume conduction models of the cochlea.
