# Correction: Parameterisation and Prediction of Intra-canal Cochlear Structures

**DOI:** 10.1007/s10439-025-03711-4

**Published:** 2025-03-20

**Authors:** Joshua Thiselton, Tania Hanekom

**Affiliations:** Bioengineering, Department of Electrical, Electronic and Computer Engineering, University of Pretoria, Lynnwood Road, Pretoria, 0002 Gauteng South Africa

**Correction to: Annals of Biomedical Engineering (2024) 52:695–706** 10.1007/s10439-023-03417-5

The funding note is incomplete. The funding note should read as follows:

## Funding

Open access funding provided by University of Pretoria. This work is based on research supported by the National Research Foundation of South Africa (Grant number 141954). The authors have no other relevant financial or non-financial interests to disclose.

